# Minimally invasive photoacoustic imaging: Current status and future perspectives

**DOI:** 10.1016/j.pacs.2019.100146

**Published:** 2019-10-31

**Authors:** Tianrui Zhao, Adrien E. Desjardins, Sebastien Ourselin, Tom Vercauteren, Wenfeng Xia

**Affiliations:** a School of Biomedical Engineering and Imaging Sciences, King’s College London, 4th Floor, Lambeth Wing St Thomas’ Hospital London, London SE1 7EH, United Kingdom; b Department of Medical Physics and Biomedical Engineering, University College London, Gower Street, London WC1E 6BT, United Kingdom; c Wellcome/EPSRC Centre for Interventional and Surgical Sciences, University College London, Charles Bell House, 67-73 Riding House Street, London W1W 7EJ, United Kingdom

**Keywords:** Photoacoustic imaging, Photoacoustic computed tomography, Photoacoustic microscopy, Multi-modal imaging, Minimally invasive procedures, Interventional photoacoustic imaging, Photoacoustic endoscopy

## Abstract

Photoacoustic imaging (PAI) is an emerging biomedical imaging modality that is based on optical absorption contrast, capable of revealing distinct spectroscopic signatures of tissue at high spatial resolution and large imaging depths. However, clinical applications of conventional non-invasive PAI systems have been restricted to examinations of tissues at depths less than a few cm due to strong light attenuation. Minimally invasive photoacoustic imaging (miPAI) has greatly extended the landscape of PAI by delivering excitation light within tissue through miniature fibre-optic probes. In the past decade, various miPAI systems have been developed with demonstrated applicability in several clinical fields. In this article, we present an overview of the current status of miPAI and our thoughts on future perspectives.

## 1 Introduction

Photoacoustic imaging (PAI), also known as optoacoustic imaging, has been one of the fastest growing fields in biomedical imaging in the last decade [[1], [2], [3], [4], [5], [6], [7]]. This hybrid imaging modality is based on the detection of light-induced ultrasound (US) waves. Under nanosecond-pulsed or temporally modulated light illuminations, specific endogenous tissue chromophores or exogenous contrast agents absorb and convert optical energy to localised and rapid rises in temperature, resulting in the generation of US waves. The generated US waves can be detected by US detectors to form images of optical absorption of the tissue chromophores or contrast agents. As such, PAI encodes rich optical contrast into US waves, revealing spectroscopic information of biological tissue that is originated from chemical composition at highly scalable spatial resolution and imaging depths. The spatial resolution of PAI ranges from sub-micrometres at sub-millimetre imaging depths to sub-millimetres at depths up to several cm [1,[8], [9], [10]]. Furthermore, multispectral PAI (also known as multispectral optoacoustic tomography) is capable of providing tissue functional information including blood oxygen saturation and metabolism with optical excitation at multiple wavelengths [[11], [12], [13], [14], [15], [16], [17]].

### 1.1 Image contrast

**Fig. 1:**
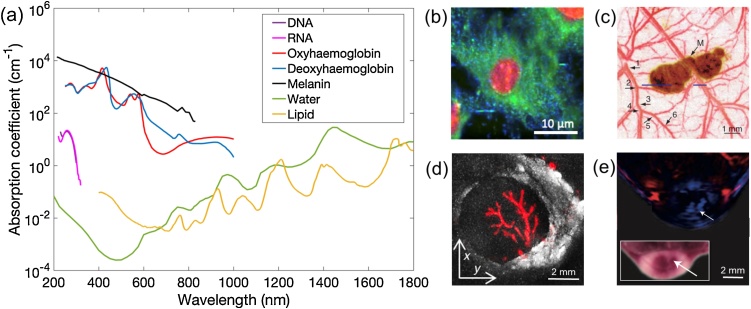
Endogenous tissue chromophores in photoacoustic imaging. (a) Optical absorption spectra of the main tissue chromophores, including DNA (data adapted from [20]), RNA (data adapted from [22]), oxyhaemoglobin, deoxyhaemoglobin (150 g L^−1^), melanin (data from https://omlc.org/spectra/), water and lipid (data adapted from [18]). (b) Ultraviolet localised photoacoustic (PA) image of a fibroblast cell with lipids, protein and nucleic acids contents shown in pseudo-coloured blue, green and red, respectively. This image was adapted from Ref [21] with permission. (c) PA image of a melanoma mouse model *in vivo*. Melanoma (pseudo-coloured brown) visualised with 784 nm excitation was surrounded by microvasculature (pseudo-coloured red) visualised at 584 nm with six orders of vessel branching. M, Melanoma. This image was adapted from Ref. [15] with permission. (d) PA image of a human lymph node with lipid (grayscale) and haemoglobin (pseudo-coloured red) contrast with optical excitation at 1210 nm and 530 nm, respectively. This image was adapted from Ref. [26] with permission. (e) Multispectral PAI of a mouse tumour model. Oxyhaemoglobin and deoxyhaemoglobin distributions were pseudo-coloured in red and blue, respectively. White arrows indicated the region of a tumour core with a low level of blood oxygen concentration. Inset is a photograph of the corresponding cross-section of the imaged tumour region. This image was adapted from Ref. [6] with permission.

Endogenous tissue chromophores provide rich optical absorption contrast in PAI with different tissues dominating light absorption in varying wavelength ranges (Fig. 1a) [[1], [2], [3], [4], [5], [6], [7], [8], [9], [10], [11], [12], [13], [14], [15], [16], [17], [18]]. For example, in the ultraviolet range from 180 nm to 400 nm, strong light absorption of DNA and RNA enables the visualisation of cell nuclei without staining [[19], [20], [21], [22]]. In the visible range from 500 nm to 600 nm, and the near-infrared range from 700 nm to 900 nm, haemoglobin is commonly targeted as the primary chromophore to provide both structural and functional information of microvasculature [[14], [15], [16]]. As oxyhaemoglobin and deoxyhaemoglobin have different absorption spectra, blood oxygen saturation can be obtained with multiwavelength measurements [[14], [15], [16]]. In the near-infrared range from 900 nm to 1800 nm, lipid is an important endogenous chromophore owing to its prominent optical absorption peaks at 930 nm, 1210 nm and 1720 nm [1,13,[23], [24], [25], [26]]. Melanin is highly localised in the skin and retina and exhibits a strong absorption over a broad range of wavelengths, enabling sensitive characterisation of melanoma cells with PAI [15,27,28].

A wide range of exogenous contrast agents have been investigated for PAI [[29], [30], [31], [32]]. Exogenous contrast agents typically comprise a signalling compound to generate US and a target ligand that reacts with the tissue of interest [29]. A number of exogenous contrast agents were studied to extend photoacoustic (PA) contrast, such as small molecular dyes, metal and carbon nanoparticles and organic nanostructures [[29], [30], [31], [32]]. Recently, genetically encoded chromophores have shown promise in studies with animal models, in which specific cell lines were genetically modified to express proteins that provided optical absorption contrast [[33], [34], [35], [36]]. While promising results have been reported in various studies, most of them involving exogenous contrast agents have been restricted to tissue-mimicking phantoms or animal models. Only a few small molecular dyes have been approved by the FDA for human use including methylene blue and indocyanine green.

With the knowledge of the optical absorption spectra, a particular light-absorbing structure can be targeted as the primary chromophore or contrast agent to provide contrast at a chosen wavelength (Fig. 1b-d) [15,21,26]. Furthermore, the spatial distributions of multiple light-absorbing structures can be recovered simultaneously using spectral unmixing algorithms with PA data acquired at multiple wavelengths (Fig. 1e) [6].

### 1.2 Photoacoustic computed tomography and microscopy

**Fig. 2:**
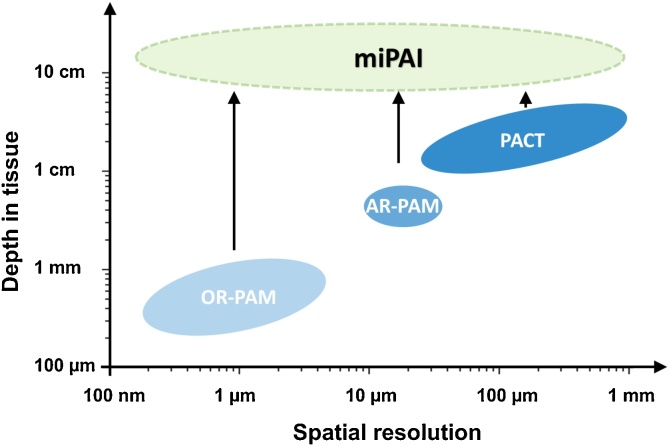
Spatial resolution versus depth of examinations for conventional and minimally invasive photoacoustic imaging (miPAI) techniques. OR-PAM, optical resolution-photoacoustic microscopy; AR-PAM, acoustic resolution-photoacoustic microscopy; PACT, photoacoustic computed tomography. For simplicity purposes, the spatial resolution of OR-RAM only represents the lateral resolution. With excitation light delivered within tissue through miniature fibre-optic probes, miPAI greatly extended the depths of examinations of conventional non-invasive photoacoustic imaging modalities including OR-PAM, AR-PAM and PACT.

PAI can be categorised as PA computed tomography (PACT) and PA microscopy (PAM, also known as raster-scan optoacoustic microscopy) based on their fundamental differences in implementation and image reconstruction [[1], [2], [3],^9^,^10^]. In conventional PACT, nanosecond-pulsed or intensity-modulated continuous-wave excitation light is delivered to illuminate tissue objects. The light then diffusively propagates through tissue and is absorbed by tissue chromophores or contrast agents, which can give rise to US waves in the MHz range. These US waves propagate outwards in all directions; some arrive at the tissue surface where they can be received by an array of US detectors, or a single-element detector scanned mechanically to act as an array. With the knowledge of the sound velocity in tissue, cross-sectional or volumetric images of the absorbing structures can be reconstructed using various algorithms, including filtered back-projection [37,38], time reversal [39], frequency-domain [40], and model-based algorithms [41]. As such, relying on diffusive light rather than focused light, the imaging depth of PACT can be as large as several cm [1,4,42]. The spatial resolution of PACT is dictated by the US detection, including the frequency response and spatial sampling steps of the US detector. Typically, the spatial resolution of PACT ranges from several tens of μm at an imaging depth of a few mm to several hundreds of μm at an imaging depth of a few cm (Fig. 2) [1,2].

Depending on the spatial resolution, PAM can be implemented in two forms: optical-resolution PAM (OR-PAM) and acoustic-resolution PAM (AR-PAM). In OR-PAM, a tightly focused light beam is raster-scanned over a 2D region of interest, whilst in AR-PAM, a focused single-element US detector is employed for scanning over a tissue target in 2D that is illuminated by a quasi-focused light beam. For both forms, a time-resolved US signal is detected by a single-element US detector at each scanning location to form a depth-resolved A-scan line, whilst multiple A-lines acquired from scanning over a 1D or 2D surface region resemble a 2D or 3D image, respectively. The lateral resolution of OR-PAM is determined by the size of the diffraction-limited optical focal spot. The imaging depth is restricted within the optical transport mean free path (optical diffusion limit), which is typically less than 1 mm in biological tissues [10]. Similarly, the lateral resolution of AR-PAM is determined by the size of the acoustic focus, which is usually a few tens of μm [1,2,3,10]. As AR-PAM is not dependent on optical focusing; its imaging depth can reach a few mm, thus breaking through the optical diffusion limit. For both OR-PAM and AR-PAM, the axial resolution driven by the time resolution of the US detection is usually between 10–100 μm, depending on the frequency response of the US detector (Fig. 2) [1,10].

### 1.3 Minimally invasive photoacoustic imaging

**Fig. 3:**
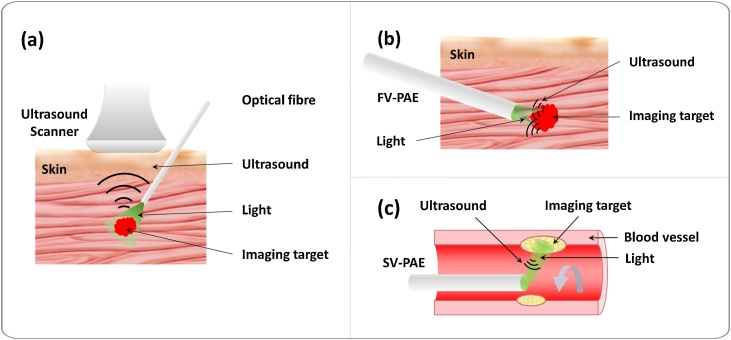
Schematic illustrations of the three embodiments of minimally invasive photoacoustic imaging (miPAI) techniques including (a) Interventional photoacoustic imaging (iPAI), (b) Forward-viewing photoacoustic endoscopy (FV-PAE), and (c) Side-viewing photoacoustic endoscopy (SV-PAE).

Numerous PAI applications have been explored in many fields in biomedical sciences with extensive clinical [23,25,26,[43], [44], [45], [46]] and pre-clinical studies [15,17,[47], [48], [49], [50], [51]], including neurology [15,17,36,47,48], oncology [25,26,[49], [50], [51]], cardiology [23,[43], [44], [45]], dermatology [27,46] and cell biology [19,20,21]. However, the maximum tissue depth at which PAI can achieve sufficient contrast from endogenous chromophores has been limited to ∼4 cm [[50], [51], [52], [53], [54]]. This is due to strong light attenuation of biological tissues. According to a calculation by Beard [1] based on physiologically realistic optical properties of tissue, light fluence at 700 nm decreases by approximately a factor of 4 with each 1 cm increment of tissue penetration depth, once beyond the first a few mm. To extend PAI applications to examining deep internal tissues, minimally invasive photoacoustic imaging (miPAI) has been an area of intensive research interests in the past few years (Fig. 2) [[55], [56], [57], [58], [59], [60], [61], [62], [63], [64], [65], [66], [67]]. In contrast to conventional non-invasive PAI, for which excitation light is delivered to the tissue surface, miPAI delivers light directly to tissue targets via an optical fibre embedded within an instrument channel of an interventional medical device. Two forms of miPAI systems have been investigated depending on if the US detection performed is outside or inside the body; the former largely represents interventional PA imaging (iPAI), and the latter, PA endoscopy (PAE). Typically, an iPAI system employs a light delivery optical fibre to excite PA signals from tissue with percutaneous insertions, and a commercial US system with an imaging array probe located at the skin surface for signal detection (Fig. 3a). Different from iPAI that can be regarded as a variant of conventional PACT, PAE is primarily based on the principle of PAM. In PAE, both the light delivery system and the US detector are usually integrated into a single miniature probe that can be inserted into the body to visualise internal organs with a forward-viewing (Fig. 3b) or a side-reviewing capability (Fig. 3c).

A number of miPAI systems have been developed for applications in a variety of clinical fields at varying development stages, including fetal medicine [63,68], cardiology [55,56,[69], [70], [71]], regional anaesthesia and pain management [72,73], and oncology [57,74,75]. The aim of this review is to provide an overview of the current implementations of miPAI systems, their development stages and potential clinical applications. The structure of this review is as follows: Section 2 gives an overview of iPAI systems and their potential clinical applications. Section 3 reviews recent developments on PAE, including forward-viewing (Section 3.1), side-viewing (Section 3.2), multimodal PAE probes (Section 3.3) and their potential clinical applications (Section 3.4). Finally, in Section 4, our thoughts on future directions of technological development for miPAI are discussed.

## 2 Interventional photoacoustic imaging

PAI has shown promise for guiding surgical and interventional procedures [[76], [77], [78], [79]], as it can provide functional and molecular information of tissue with high spatial resolution in real-time. Park et al. [76] proposed a fusion imaging method that co-registers and overlays pre-acquired MR and real-time PA/US images for diagnosis and therapy monitoring. Lee et al. [77] recently developed a dual-modal system based on a conventional surgical microscope that combined PAI and optical coherence tomography (OCT) for intraoperative surgical guidance. These approaches are likely to be useful for visualising sub-surface tissue structures with PAI during surgical procedures. However, many surgical and interventional procedures require the visualisation of procedure targets at depths that are well beyond the imaging depths of conventional PAI. To address this challenge, iPAI has been an area of intensive research in the last few years as a promising tool for guiding minimally invasive procedures [[62], [63], [64],^68^,[72], [73], [74], [75],[80], [81], [82], [83], [84], [85], [86]].

### 2.1 Interventional photoacoustic imaging systems

US imaging is widely used for guiding minimally invasive procedures as it can provide anatomical information with high spatial resolution in real-time and is easily assessible. However, US imaging suffers from low soft tissue contrast which makes it sometimes challenging to differentiate tissue targets and interventional medical devices from surrounding tissue. PAI holds great potential to overcome this limitation by providing molecular information of tissue based on rich optical absorption contrast. In most iPAI systems (Fig. 3a), a clinical US imaging system is used to perform naturally co-registered, interleaved US and PA imaging that shares the same probe for US detection. As such, iPAI is capable of revealing both tissue anatomical information with US imaging and molecular information with PAI. The configuration of the light delivery remains the major difference between conventional PACT and iPAI, in which the excitation light is usually delivered through an optical fibre that can be integrated with an interventional medical device to directly illuminate the tissue targets [[72], [73], [74], [75],^81^]. In contrast to conventional non-invasive PACT which suffers from rapid decreasing of signal strength with increasing tissue depth due to strong light attenuation in tissue, iPAI usually allows for greater imaging depths of several cm [82]. Various iPAI systems are summarised in Table 1.

**Table 1:** Representative interventional photoacoustic imaging systems. L, lateral; A, axial.

Reference	Light delivery probe	Laser source	Ultrasound system	Spatial Resolution (μm)	Targeted application	Validation
Piras et al. (2013) [62]	600-μm-diameter optical fibre in a biopsy needle	1064 nm, 250 Hz (Diny pQ, IB laser)	7.5 MHz, 128 elements (L10-5, Picus, Esaote Europe BV)	–	Breast biopsy guidance	Breast phantom with fish heart as inclusions
Kruizinga et al. (2014) [81]	400-μm-core-diameter, side-firing fibre in a 1.25 mm rigid steel tube	1130 - 1250 nm (OPOTEK Vibrant B/355-II)	8 MHz, 256 elements (ATL 12L5 + Verasonics V1)	–	Carotid artery atherosclerosis	Human carotid artery *ex vivo*
Xia et al. (2015) [63]	910-μm-cores-diameter optical fibre in a needle	750 - 900 nm, 1150 - 1300 nm, 10 Hz (VersaScan L-532, GWU-Lasertechnik)	5 - 14 MHz, 128 elements (L14-5/38, SonixMDP, Analogic Ultrasound)	L: 600 - 1000 A: ∼100	Fetal surgery and nerve blocks guidance	Human placenta ex vivo + nerve and vessel phantom (porcine fat and human blood in tubes)
Bell et al. (2015) [80]	1-mm-core diameter optical fibre attached to a surgical tool	1064 nm	5-14 MHz (L14-5W/60, Ultrasonix)	–	Endonasal Surgeries	Bone and blood vessel phantom
Bell et al. (2015) [82]	1-mm-core diameter, side-firing optical fibre in a 2 mm quartz tube	1064 nm, 10 Hz (Phocus InLine, Opotek)	4 - 8 MHz linear array + 5 - 9 MHz curvilinear array (BPL9-5 + BPC8-4, Ultrasonix + SonixTouch, Ultrasonix)	–	Prostate cancer detection and treatment	Brachytherapy seeds
Singh et al. (2016) [85]	600-μm-diameter, side-firing optical fibre in a biopsy needle	720 - 860 nm, 10 Hz (Quanta-Ray Pro 250, Spectra Physics, VersaScan-L532, GWU)	7.5 MHz, 128 elements (SL3323, MyLab_One, Esaote Europe BV)	–	Prostate cancer detection and treatment	Brachytherapy seeds
Allard et al. (2018) [84]	Fibres attached to a da Vinci® scissor tool	1064 nm	Alpinion L3-8, Alpinion ECUBE 12R	–	Teleoperated hysterectomies	Blood vessel phantom
Li et al. (2018) [86]	1.5-mm-core-diameter optical fibre fused with a 3-cm-long active diffuser made of silica	1064 nm (Q-smart 850, Quantel Laser)	5 MHz, 128 elements (L7-4, Vantage 128, Verasonics)	L: 931 A: 419	Cancer screening and intraoperative guidance	Mouse *in vivo*

### 2.2 Interventional imaging of procedure targets

**Fig. 4:**
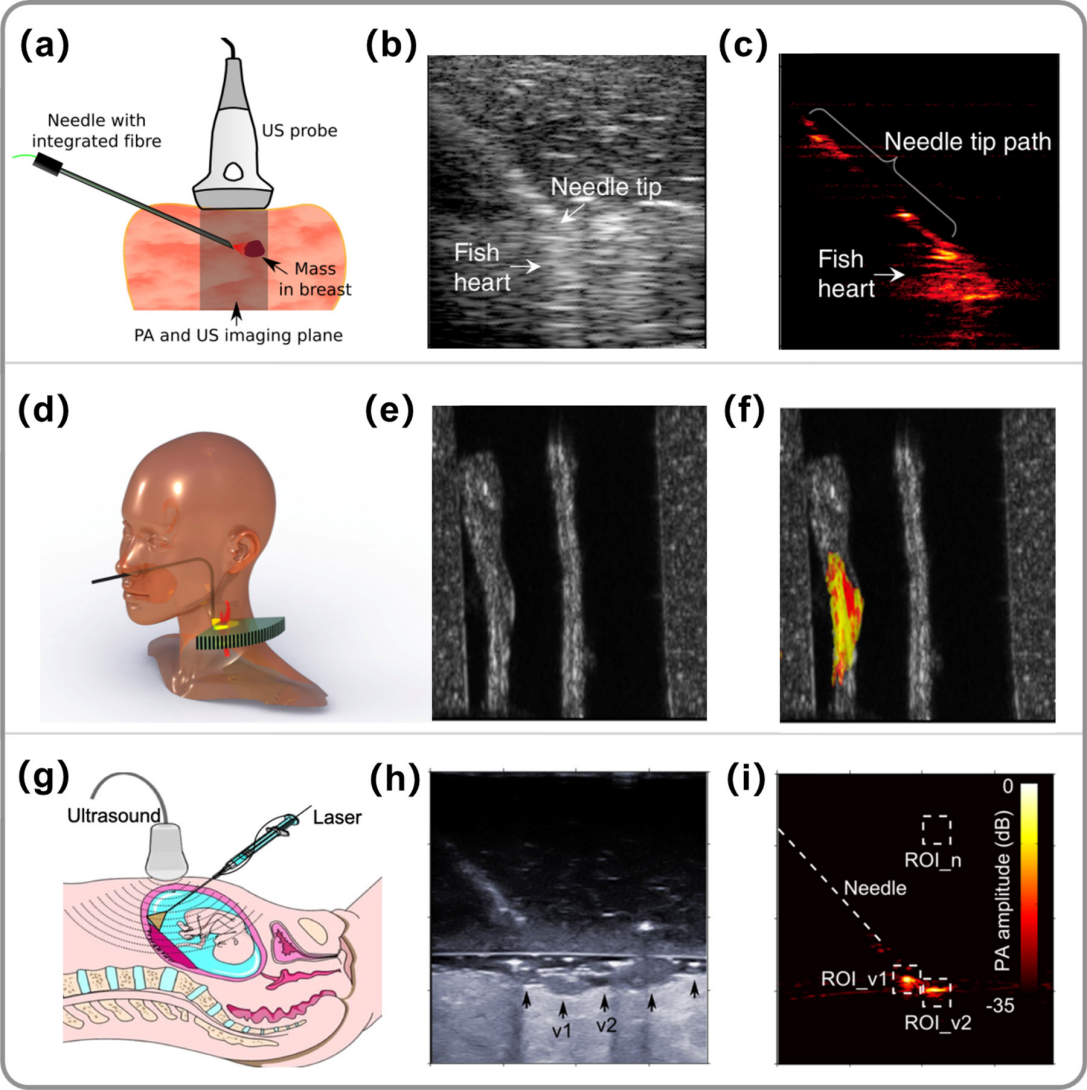
Embodiments of interventional photoacoustic imaging (iPAI) systems and their targeted clinical applications. (a) Schematic diagram of an iPAI system for guiding breast biopsy, in which the excitation light (1064 nm) was delivered through an optical fibre embedded in a breast biopsy needle and photoacoustic (PA) signals were detected by a clinical ultrasound imaging probe. To demonstrate the concept, (b) Ultrasound (US) and (c) PA images were obtained during needle insertions towards a tumour-mimicking target (fish heart). (b)-(c) were adapted from Ref. [62] with permission. (d) Schematic diagram of an iPAI system for the diagnosis of carotid artery atherosclerosis. Excitation light was proposed to be delivered transnasally via a side-firing optical fibre to illuminate the carotid artery in the pharynx cavity, while US detection used an external linear array transducer placed at the neck side. The system was validated with an *ex vivo* diseased human carotid artery embedded in a neck phantom. Co-registered grayscale US (e) and colour-coded PA (f) images demonstrated complementary information, with US visualising anatomical structure and PA revealing the lipid composition of the plaque. (d)-(f) were adapted from Ref. [81] with permission. (g) Schematic diagram of a multispectral iPAI system for guiding the treatment of twin-to-twin transfusion syndrome, in which the light was delivered through the working channel of a fetoscope via an optical fibre to visualise the placenta vasculature. US detection was performed by an external linear array US probe at the abdomen. This concept was demonstrated with a freshly excised human twin placenta. An agar block was placed between the US probe and the placenta to mimic the ammonitic fluid. US images (h) revealed the anatomical structure of the placenta; a few surface blood vessels were barely visible. In contrast, PA images (i) clearly visualised two blood vessels (v1 and v2) under illumination. (h)-(i) were adapted from Ref. [63] with permission.

A number of iPAI systems have been developed for various clinical applications in recent years. In 2013, Piras et al. developed an iPAI system for the first time for breast biopsy guidance by delivering pulsed laser at 1064 nm into tissue through an optical fibre embedded in a biopsy needle (Fig. 4a-c) [62]. Promising results were obtained with tissue-mimicking phantoms by obtaining PA contrast based on the optical absorption of haemoglobin. In 2014, Kruizinga et al. [81] proposed an iPAI system for the diagnosis of carotid artery atherosclerosis by characterising lipid content in vulnerable plaques (Fig. 4d–f). With this system, a 1.25-mm-diameter, side-firing optical probe was proposed to be inserted into the human pharynx to internally illuminate the carotid artery wall. This concept was validated by imaging a tissue-mimicking phantom and a post-mortem common carotid artery. In 2015, Xia et al. [63] reported the development of a multispectral iPAI system using excitation light at a wide range of wavelengths for guiding minimally invasive procedures including fetal surgery (Fig. 4g-i) [63,68] and peripheral nerve blocks [72,73]. Validation was performed on *ex vivo* human placentas [63,68], *ex vivo* [72] and *in vivo* [73] swine peripheral nerves. In 2015, Bell et al. [80] proposed an iPAI system for guiding transnasal neurosurgery, in which optical fibres were attached onto a surgical tool that could be inserted into the nasal passage to illuminate the sphenoid bone to visualise blood vessels and the sphenoid bone, while US detection could be based on an US transducer placed on the temporal region. In 2017, Gandhi et al. [83] proposed an iPAI system, in which an optical fibre was integrated onto a da Vinci surgical arm for the guidance of surgical procedures by quantifying the separation among blood vessels and nerves.

### 2.3 Interventional imaging of medical devices

In additional to visualising tissue targets, iPAI was also proposed to visualise interventional medical devices during minimally invasive procedures. In 2014, Bell et al. [64] designed a transurethral light delivery system by inserting an optical fibre into a urinary catheter to image prostate brachytherapy seeds during prostate brachytherapy and validated it on a canine prostate *in vivo* [64,82]. In 2018, Allard et al. [84] investigated the potential of iPAI for the guidance of hysterectomies, in which excitation light was delivered through a fibre bundle that surrounded a curved scissor tool of a surgical robot to illuminate both the tissue targets and the surgical tool.

### 2.4 Current challenges

Although the reported iPAI systems in literature so far have been promising, there are a few limitations associated with the interventional light delivery approach. First, the light delivered from the optical fibre has a highly non-uniform fluence distribution in tissue, which makes fluence compensation for accurate recovery of chromophore distributions nontrivial. Second, due to the non-uniform fluence distribution, PA signals generated at the fibre tip can sometimes lead to off-plane artefacts when the fibre tip is outside the imaging plane [64]. As such, interpretation of PA images can be challenging. Finally, the strong PA signals generated right at the fibre tip can be reflected by nearby interventional devices causing image artefacts. Efforts have been made by a few research groups to address these limitations. In 2016, Singh et al. [85] reported a PA-guided focused US method to identify and remove this type of image artefacts in an iPAI system. In 2018, Li et al. [86] fused a needle-shaped silica diffuser at the fibre tip to achieve a relatively homogenous light illumination pattern.

## 3 Photoacoustic endoscopy

The design of a PAE probe usually involves combining a miniature US detector with an optical fibre that delivers excitation light to obtain PA images of internal tissue structures at micron-scale spatial resolution. Various PAE probes, including forward-viewing and side-viewing PAE probes, as summarised in Table 2, have been developed in the last decade for different clinical applications.

**Table 2:** Representative photoacoustic endoscopy systems. FV, forward-viewing; SV, side-viewing; fps, frames per second; λ, wavelength; f-3 dB, -3 dB frequency bandwidth; OR, optical-resolution; AR, acoustic-resolution; L, lateral; A, axial; PA, photoacoustic; US, ultrasound; OCT, optical coherence tomography.

Reference	View	Modality	Resolution mode	Light source	Ultrasound sensor	Probe diameter (mm)	Resolution (μm)	Imaging speed (fps)	Validation
Shao et al. (2012) [87]	FV	PA + Fluorescence	OR	532 nm, 160 kHz (GLP-10, IPG Photonics Corporation)	Piezoelectric, Focused, 3.5 MHz	0.85	L: ∼7	2	Mouse ear *in vivo*
Papadopoulos et al. (2013) [67]	FV	PA	OR	532 nm, (NL-201, EKSPLA)	Piezoelectric, Focused,20 MHz	0.22	L: 1.5	–	Nylon wire phantom
Stasio et al. (2015) [90]	FV	PA + fluorescence	OR	532 nm, 200 Hz (NL-201, EKSPLA, Lithuania)	Piezoelectric, Focused,20 MHz	0.33	L: 8	–	Nylon thread phantom
Ansari et al. (2018) [66]	FV	PA	AR	410 - 2100 nm, 30 Hz (Innolas Spitlight 600)	Fabry-Perot resonator, f_-3dB_ = 34 MHz, λ/4 resonance at 21 MHz, λ/2 minimum at 67 MHz	3.2	L: 31, A: 45-170	–	Duck embryo + Mouse skin *ex vivo*
Caravaca-Aguirre et al. (2018) [92]	FV	PA + fluorescence	OR	532 nm, 7 kHz (Cobolt TorTM series)	Fabry-Perot resonator, 250 kHz - 50 MHz	0.25	–	–	Red blood cells + Absorbing micro-structure
Jansen et al. (2011) [56]	SV	PA + US	AR	715 - 1800 nm, 10 Hz (OPOTEK Vibrant B/ 355-II)	Piezoelectric, Unfocused, 30 MHz	1.25	–	–	Human coronary *ex vivo*
Karpiouk et al. (2012) [127]	SV	PA + US	AR	1064 nm, 20 Hz (Polaris II, New Wave, Inc.)	Piezoelectric, Unfocused, 40 MHz	2.2	–	–	Rabit artery + Stent *in vivo*
Yang et al. (2012) [57]	SV	PA + US	AR	562 nm + 584 nm/523 nm + 640 nm, (Cobra HRR, Sirah, INNOSLAB IS811-E, EdgeWave)	Piezoelectric, Focused,36 MHz	3.8	–	4	Rabbit esophagus + Rat colon *in vivo*
Dong et al. (2014) [109]	SV	PA	OR	532 nm (TLB-6712, New Focus)	Micro-ring resonator, 5 MHz − 1 GHz	4.5	L: 4.5 A: 16	–	Plastic tube phantom
Abran et al. (2014) [117]	SV	PA + US + Fluorescence	AR	710 nm, 20 Hz (Quanta-Ray INDI series, Newport Corporation)	Piezoelectric, Unfocused, 45 MHz	1.4	–	–	Blood-mimicking phantom
Li et al. (2015) [101]	SV	PA + US	AR	1185-1235 nm, 1 kHz (EKSPLA, NT242)	Piezoelectric (Lead zirconate titanate composite), Unfocused, 40 MHz	0.9	L: ∼ 180 A: ∼ 100	5	Stent + Porcine aorta *ex vivo*
VanderLaan et al. (2016) [128]	SV	PA + US	AR	1064 nm, 10 kHz (SOL40W-1064, RPMC Lasers, Inc.)	Piezoelectric, Unfocused, 40 MHz	1	–	33	Stent phantom
He et al. (2016) [105]	SV	PA	OR + AR	532 nm, 2 kHz (Wedge HB532, BrightSolutions SRL)	Piezoelectric, Unfocused, 20 MHz	3.6	L: 13 (OR) ∼250 (AR)	–	Mouse ear *ex vivo*
Dai et al. (2017) [116]	SV	PA + US + OCT	OR	710 - 1210 nm, 20 Hz	Piezoelectric, Unfocused, 40 MHz	1	–	–	Mouse ear + Human hand *in vivo* + human arteries *ex vivo*
Wu et al. (2017) [126]	SV	PA + US	AR	1700 - 1750 nm, 5 kHz (FQ-OPO, Elforlight Ltd)	Piezoelectric, Unfocused, 40 MHz	1.3	–	20	Swine coronary arteries *in vivo* + Human coronary artery *ex vivo*
Hui et al. (2017) [104]	SV	PA + US	AR	1700 nm, 2 kHz	Piezoelectric, Unfocused, 40 MHz	1	L: 305	16	Human coronary artery *ex vivo*
Mathews et al. (2018) [111]	SV	PA + OCT	OR	560 - 610 nm, 2.8 kHz (Elforlight)	Fabry-Perot resonator, −6 dB bandwidth: 3–30 MHz	1.25	L: 18 - 40 A: ∼45	–	Stent + Vascular phantom
Liu et al. (2018) [118]	SV	PA + Hyperspectral	OR	532 nm, 10 kHz (Model DTL-314QT)	Piezoelectric, Focused,10 MHz	12	L: 40 A: 60	–	Rabbit rectum *in vivo*
Li et al. (2019) [131]	SV	PA + US	AR	532 nm, 300 kHz (DCH-532-10, Photonics Industries International Inc.)	Piezoelectric, Unfocused, 45 MHz	1	L :250 A: 50	50	Rat rectum *in vivo*

### 3.1 Forward-viewing PAE probes

The capability of forward-viewing is important for PAE in many minimally invasive procedures such as tumour biopsy, fetal and laparoscopic surgeries. The main challenges involved in the development of forward-viewing PAE probes are associated with the integration of both the light delivery and US detection units within a miniature probe. The former was achieved by rapidly scanning an optical focus either through a coherent multicore fibre bundle [65,[87], [88], [89]] or through a multimode optical waveguide via wavefront shaping [67,90,91] or by speckle illumination with compressed sensing schemes [92]. The latter requires highly miniaturised US detectors with sufficient sensitivity and frequency bandwidths.

**Fig. 5:**
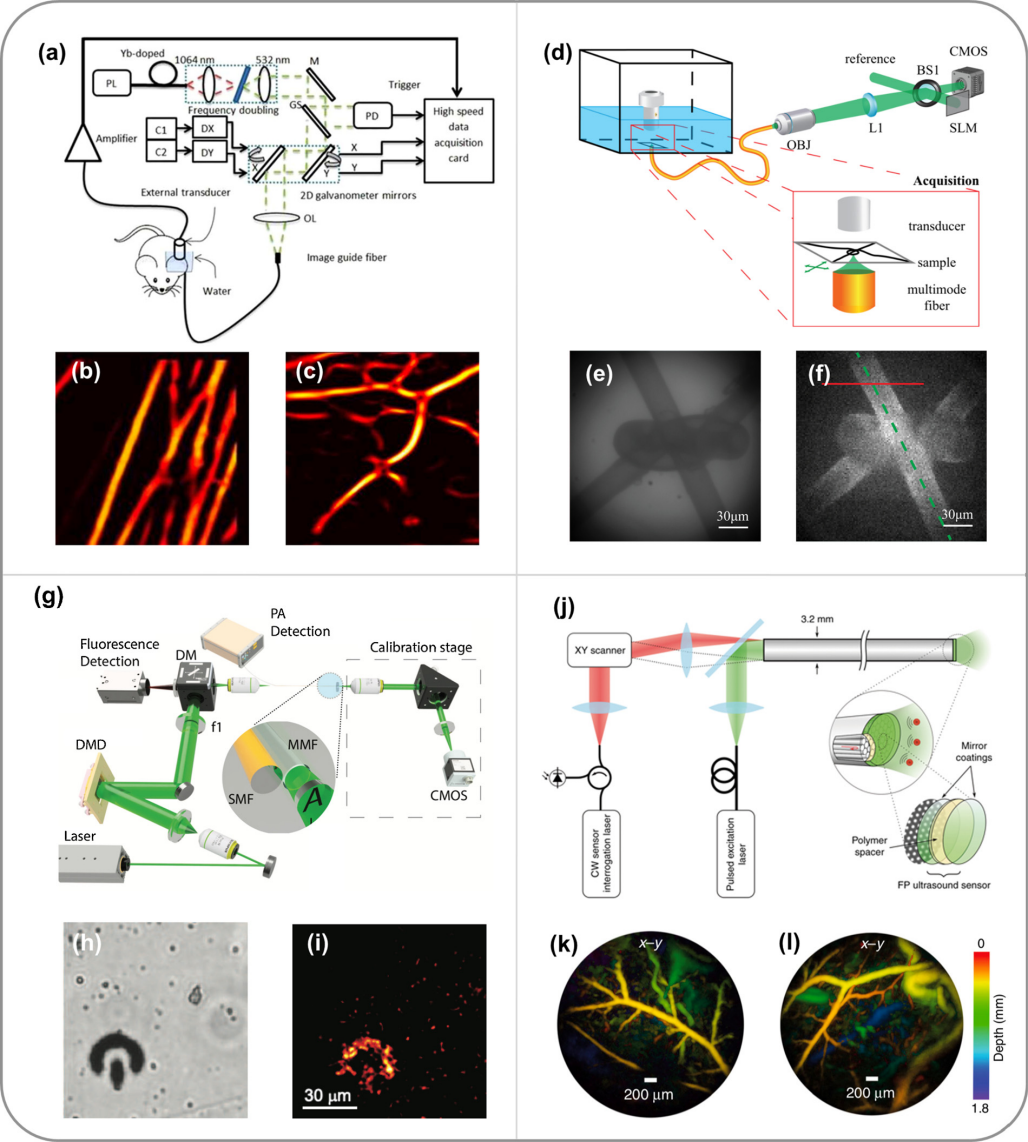
Embodiments of forward-viewing photoacoustic endoscopy (PAE) systems. (a) Schematic diagram of an optical-resolution PAE system based on a multi-core coherent fibre bundle and 2D galvanometer mirrors. Excitation light was focused at the proximal end of the fibre bundle and raster-scanned by the galvanometer mirrors for photoacoustic (PA) excitation. M, mirror; GS, glass; PD, photodiode; C1, C2, controllers; DX, DY, X and Y axis mirror drivers; OL, objective lens. (b) PA image of carbon fibre network. (c) PA image of the microvascular of a mouse ear. (a)-(c) were adapted from Ref. [65] with permission. (d) Schematic diagram of an optical-resolution PAE system based on a multimode optical fibre and a spatial light modulator (SLM). After calibration, the SLM modulated the incident light field to focus light through the multimode fibre for PA excitation. CMOS, complementary metal oxide semiconductor camera; BS1, beam splitter; L1, tube lens; OBJ, objective. (e) White light optical image and (f) PA image of a wire knot. (d)-(f) were adapted from Ref. [67] with permission. (g) Schematic diagram of an optical-resolution PAE system based on a multimode optical fibre and speckle illuminations. Pre-recorded speckle patterns were generated by a digital micro-mirror device at the distal end of the fibre for PA excitation. A model-based algorithm was used for image reconstruction. f1, tube lens; SMF, single-mode fibre; MMF, multimode fibre; CMOS, complementary metal oxide semiconductor camera. (h) Bright-field microscopy and (i) PA images of an absorbing micro-structure. Scale bar, 30 μm. (g)-(i) were adapted from Ref. [92] with permission. (j) Schematic diagram of an acoustic-resolution PAE system that is based on a multi-core coherent fibre bundle with a Fabry-Pérot (FP) cavity at its distal end to serve as a 2D array of ultrasound detectors. (k-l) PA images of the mouse abdominal skin microvasculature. (j)-(l) were adapted from Ref. [66] with permission.

In 2011, Hajireza et al. [65] employed a coherent fibre bundle consisting of 30,000 single-mode fibre cores for light delivery, whilst a galvanometer mirror system was used to scan a focused laser beam at the proximal end of the fibre bundle. A single-element US transducer was placed separately at the skin surface for US detection (Fig. 5a). This probe enabled PAI at the fibre distal end at a speed of 2 frames per second (fps). The microvasculature of a mouse ear was visualised *in vivo* at an optically-defined lateral resolution of 7 μm (Fig. 5b-c), with a field-of-view of 800 μm determined by the fibre diameter. Based on this design, the same group have then reported a couple of new generation systems, including a dual-modal system that combined fluorescence and PA endomicroscopy [87], systems that used an external linear array transducer to guide the probe insertion in US mode [88], and a system in which a gradient-index (GRIN)-lens was attached to the distal end of the fibre bundle to extend the working distance to ∼2 mm in front of the fibre tip [89].

Multimode fibres have been attractive for delivering the excitation light in a forward-viewing PAE system owing to their small dimensions and low costs. However, focusing light through multimode fibres were extremely challenging due to mode dispersion and mode coupling. Recently, wavefront shaping was used to focus light through multimode light waveguides in forward-viewing OR-PAE systems [67,90,91]. In these systems, a digital phase conjugate method [67] was developed to calibrate a multimode fibre; first, a focused laser beam was mechanically scanned across the distal end of the fibre, whilst the output light fields at the proximal end of the fibre were recorded holographically by a camera with an optical reference arm. The phases of the output fields were recovered from the captured holograms using a digital reconstruction method. A spatial light modulator was used to project phase conjugated light fields onto the proximal end of the fibre to form tightly focused light spots at the distal end in a time-reversed manner. Using this approach, in 2013, Papadopoulos et al. [67] developed a forward-viewing PA endomicroscopy system based on a multimode fibre with a diameter of 220 μm (Fig. 5d), which provided a lateral resolution of 1.5 μm that was determined by the size of optical focus. However, the US detector used in this system was bulky and was separated from the light delivery fibre, which is not optimum for a clinical endoscopy setting. To address this limitation, Stasio et al. [90] employed a dual-modal capillary waveguide consisting of silica cladding and a water-filled core for both delivering excitation light and collecting US signals. Laser scanning across the imaging fibre tip was achieved using a digital phase conjugate approach via the silica cladding, whilst the generated US signals propagated through the water-filled core and were detected by an US transducer at the proximal end of the waveguide. The high-resolution capability of this system was demonstrated by imaging phantoms with embedded nylon wires [90,91].

Single multimode fibre has also been studied for PAE with speckle illuminations and compressed sensing. In 2019, Caravaca-Aguirre et al. [92] reported an ultra-thin probe comprising a multimode fibre and a fibre-optic US detector (Fig. 5g). In this work, the spatial intensity distribution of the incident excitation light field was modulated by a light modulator and coupled into the proximal end of the fibre to provide distinct output speckle patterns for PA excitation. These pre-recorded speckle patterns together with received PA signals were fed to a model-based algorithm for PA image reconstruction. The performance of this probe was demonstrated by imaging of red blood cells and an absorbing micro-structure. However, while these wavefront-modulation-based PAE probes exhibit advantages of small size and high resolution, their performances are very sensitive to deformations and movements of the optical waveguides that are associated with the changes of their optical transfer functions. As a result, flexible waveguides can provide weakened or even destroyed light focusing, leading to degradation of PA image quality.

In addition to advances in novel light delivery systems, tremendous efforts have been made to develop miniature US detectors in PAE. In general, current forward-viewing PAEs are based on fibre-optic Fabry-Pérot (FP) detectors, which comprise a FP cavity at the distal end of a single-mode optical fibre [92,93]. Briefly, a FP cavity comprises a thin film of polymer sandwiched by two mirrors that are interrogated with a wavelength-tuneable CW laser. Changes in reflectivity of the FP cavity measure the impinging US pressure waves which modulate the thickness of the cavity [94]. The FP cavity can also be miniatured and integrated onto the tip of a single-mode fibre with high detection sensitivity, large bandwidth and wide acceptance angle, which is suitable for the use in endoscopic PA and US imaging systems [95,96]. Furthermore, it can be integrated with a coherent fibre bundle to serve as an US detector array. In 2018, Ansari et al. [66] developed a new class of forward-viewing PAE systems that are based on the principle of PACT (Fig. 5j). One of these systems consisted of a coherent multicore fibre bundle with a FP cavity at its distal end, with each fibre core interrogated to serve as an individual US detector element with optically-defined active surface area [66]. The FP cavity comprised two dielectric mirrors that were highly reflective (90%) between 1400 and 1600 nm and allowed the transmission of PA excitation light at 1064 nm. With the time-resolved PA signals received by the FP detector which was equivalent to a 2D array of US detector elements, 3D PA images were achieved with tomographic reconstruction [66]. The system had a field-of-view of 3.5 mm × 7 mm with a uniform axial resolution of 31 μm and the lateral resolution ranged from 40 μm at a depth of 1 mm to 175 μm at a depth of 7 mm. Microvasculature anatomy of a duck embryo (Fig. 5k) and the mouse skin (Fig. 5l) were visualised to a depth of approximately 2 mm. The imaging speed of this system was limited by the low pulse repetition frequency (PRF, 30 Hz) of the PA excitation laser, resulting in an acquisition time of ∼25 min per image. Thus, further improvement on the imaging speed is required before measurements in a clinical setting.

### 3.2 Side-viewing PAE probes

Side-viewing PAE has been a popular modality in the past decade for internal imaging of tissues with hollow structures such as arteries [56,58,59] and gastrointestinal tracts [57,75]. Side-viewing PAE probes are typically based on an optical fibre that performs circumferential-scanning for delivering the excitation light and an integrated single-element US detector for US detection. Volumetric images can be obtained with additional pullback-scanning of the probe [[56], [57], [58], [59], [60], [61]]. Co-registered PA and US images are usually acquired simultaneously with the two modalities sharing the same US detector.

Similar to PAM, side-viewing PAE probes in general can be divided into two groups: side-viewing AR-PAE and side-viewing OR-PAE. With AR probes, excitation laser is usually delivered directly through a multimode fibre. As such the acoustic focus is typically smaller than the diffusive light beam and hence mainly determines the lateral resolution. Imaging depths range from 1 to 3 mm [56,57,75]. Focused [97,98] or dual-element US transducers [99] are used to improve the lateral resolution to several tens of μm. With OR probes, a GRIN-lens is usually integrated onto the distal end of a single-mode or a multimode fibre to provide an optical focus, achieving a superior lateral resolution up to several μm [61,75]. However, the imaging depth of OR-PAE is typically less than 1 mm, restricted by the optical transport mean fee path. The axial resolution for both OR-PAE and AR-PAE probes is determined by the frequency response of the US transducer for both PA and US imaging modes. Conventionally, piezoelectric transducers with a high frequency of 20–80 MHz are used. This frequency range results in a high axial resolution that is around several tens of μm [[56], [57], [58], [59], [60], [61]]. It is also noteworthy that this value showed degradation in biological tissues due to strong acoustic attenuation at high frequencies.

**Fig. 6:**
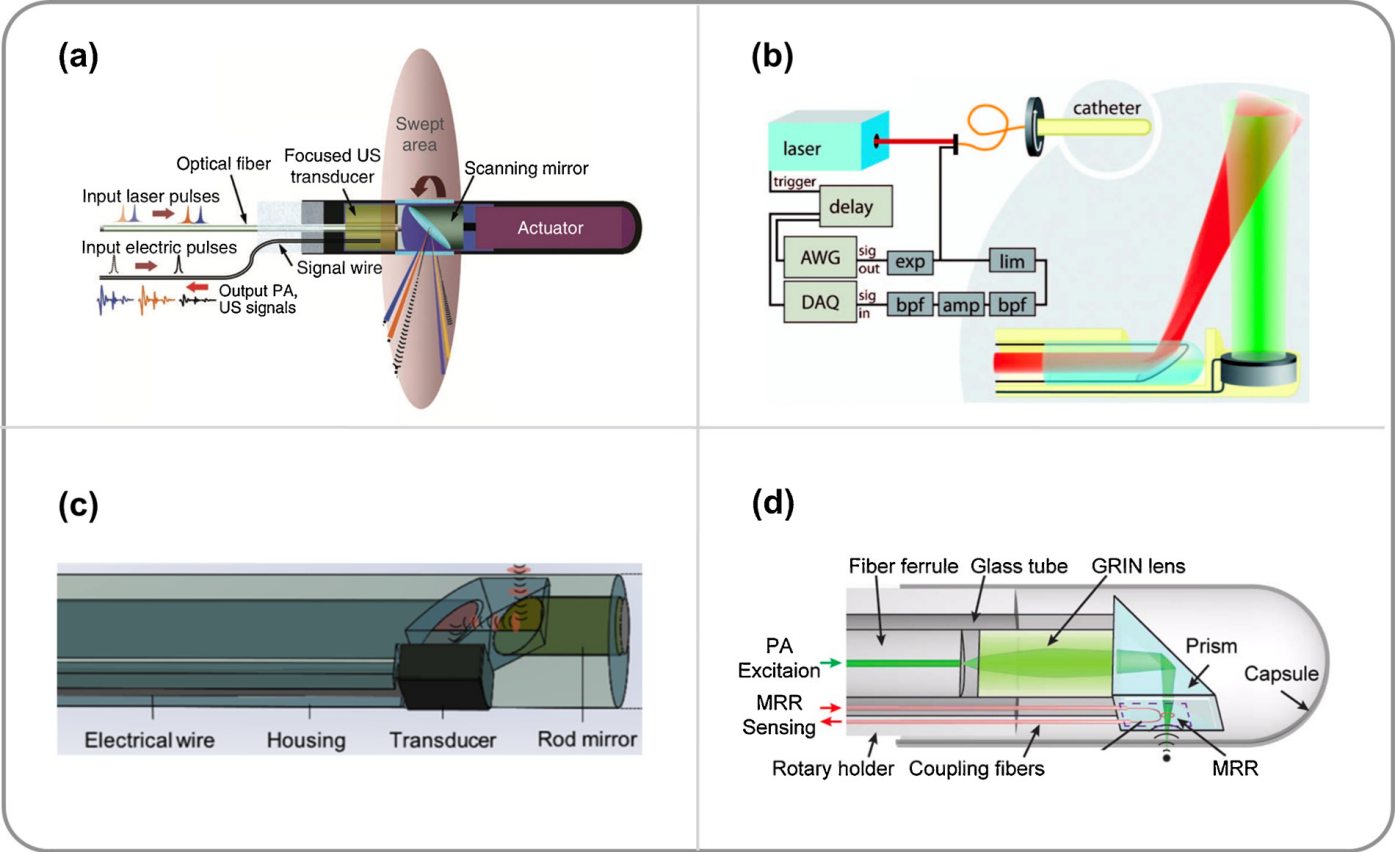
Schematic diagrams of embodiments of side-viewing photoacoustic endoscopy (PAE) probes. (a) An acoustic-resolution PAE probe with a colinear design. A ring-shaped ultrasound (US) transducer was integrated with an optical fibre, and a mirror was placed in front of the fibre to deflect both light and US. This image was adapted from Ref. [57] with permission. (b) An acoustic-resolution PAE probe with a non-colinear design. The distal end of a multimode fibre was angle-polished at 34° for side-way illuminations, and an US transducer was placed in front of the fibre tip facing the illuminated region. AWG, Arbitrary wave generator; DAQ, data acquisition, exp, expander; lim, limiter; bpf, bandpass filter, and amp, amplifier. This image was adapted from Ref. [56] with permission. (c) An acoustic-resolution PAE probe with a compact colinear design. The distal end of a multimode fibre was polished to 47° to deflect generated photoacoustic signals to an US transducer. This image was adapted from Ref. [104] with permission. (d) An optical-resolution PAE probe with a compact colinear design and a micro-ring resonator US detector. This image was adapted from Ref. [109] with permission.

Various probe configurations have been investigated. In 2009, Yang et al. [100] reported the development of a side-viewing PAE probe, in which a ring-shaped US transducer was colinearly aligned with the optical fibre. In the following years, the same group developed a series of side-viewing PAEs with the same colinear design but different components including a focused US transducer (Fig. 6a) [57,97,98] or a GRIN-lens [75] to improve the spatial resolution. Validation of imaging probes were demonstrated by imaging of the gastrointestinal tracts of small animals *in vivo*. The size of the probe was reduced to 2.5 mm in diameter compared to previous designs. However, a diameter of around 1 mm is desired for intravascular applications to avoid damaging tissues. In 2010, Karpiouk et al. [55] reported two side-viewing PAE designs employing an angle-polished fibre end or a mirror for sideway laser delivery, and a side-facing US transducer placed in front of the fibre end. In these designs, the acoustic axis of the US transducer was perpendicular to the optical axis of the fibre (non-colinear), so that the diameter of the probe can be reduced. A number of side-viewing PAE probes with the non-colinear design have been developed. In 2011, Jansen et al. [56] reported the development of a 1.25-mm-diameter PAE probe, in which an US transducer with a diameter of 1 mm was placed in front of a 34°-angle-polished fibre and facing the light-illuminated region (Fig. 6b). The small diameter of the probe allowed it to be used in human artery *ex vivo* to visualise vulnerable plaques in atherosclerosis. In 2015, Li et al. [101] reported a 0.9-mm-diameter PAE probe. However, as the light path and US transducer were arranged non-colinearly, the regions of the light illumination and the US detection were only partially overlapped (only tissues in the overlapped region can be imaged), resulting in a curved PAI plane and a large difference between the lateral and longitudinal (along the pullback direction in 3D imaging) resolution [102]. In 2016, Cao et al. [103] developed a probe that combined advantages of both colinear and non-colinear designs. This was achieved by using a mirror placed in front of a 45°-polished fibre as both an acoustic and an optical reflector, and using the polished fibre tip to reflect acoustic waves to the transducer (Fig. 6c). As a result, whilst the acoustic and light fields were co-linearly overlapped, the transducer’s normal direction was perpendicular to optical axis of the fibre, the diameter of the probe was reduced to 1 mm [104]. In addition, efforts have also been made to combine the advantages of both OR- and AR-PAE. In 2016, He et al. [105] reported a hybrid OR- and AR-PAE probe, which employed a single-mode optical fibre with a GRIN-lens at its distal end for delivering light in the OR mode and a 400-μm-diameter multimode fibre in the AR mode.

Microelectromechanical systems (MEMS) were also employed for laser scanning in side-viewing PAE probes to achieve fast 3D imaging over a cubic volume of interest, in a manner similar to that of OR-PAM [106,107]. Different from conventional probes that provide circumferential-scanning of tissue, the MEMS mirror reflected and raster-scanned the laser beam [106,107], and hence allowed planar scanning of tissue. In 2017, Guo et al. [106] reported a side-viewing PAE with a MEMS arranged in parallel with a GRIN-lens-integrated optical fibre, which achieved high-resolution (lateral resolution of 10.6 μm, axial resolution of 105 μm) PAI as demonstrated with a mouse ear and colon *ex vivo*. In 2018, Qu et al. [107] developed a MEMS-based PAE probe using a ring-shaped US transducer to detect US waves. PAI with high-resolution (lateral resolution of 3.1 μm, axial resolution of 46.5 μm) was achieved on *ex vivo* human cervix, uterine body, sublingual mucosa and *in vivo* cervical vasculatures in pregnant women. However, the use of MEMS in PAE probes led to large probe diameters (6 mm in [106] and 20 mm in [107]), and thus further reduction on the diameters of the probes is required for applications in smaller lumens such as the human arteries.

Recently, optical US detectors have been used in side-viewing PAE probes [[108], [109], [110], [111]]. In 2010, Hsieh et al. [108] designed a PAE probe with a micro-ring resonator US detector attached onto the side wall of a light delivery optical fibre. A micro-cone mirror was placed in front of the multimode fibre tip for 360° illumination, whilst a ring-shaped US transducer was integrated with the fibre for US transmission. In addition to PAI, these US transmissions were received by the micro-ring resonator for pulse-echo US imaging. In 2014, Dong et al. [109] developed an all-optical OR-PAE probe based on a polymeric micro-ring resonator for US detection and a GRIN-lens integrated at the distal end of a single-mode optical fibre for PA excitation (Fig. 6d). With phantom imaging, the lateral and axial resolution of the system was measured as 4.5 and 16 μm, respectively [109]. FP sensors have also been studied for use in side-viewing PAE probes. In 2011, Zhang *el al.* [110] proposed a single-fibre design with a dichromatic FP cavity coated on a dual-cladding optical fibre. The PA excitation light was coupled into the inner cladding of the fibre and the output was reflected by a micro-prism attached to the distal end of the fibre to provide side-way illuminations, while the single-mode fibre core was used to deliver the interrogation light to the FP cavity and collect the reflectance for US detection. In 2018, Mathews et al. [111] developed a dual-fibre probe for concurrent PAE and OCT, with a single-mode optical fibre for PA excitation light delivery and a fibre-optic FP sensor for US detection. The same single-mode optical fibre was used for performing OCT imaging.

A side-viewing PAE probe is usually encapsulated in a protective sheath for tissue imaging. The choice of the sheath material has significant impact on the system performance. An ideal sheath material for PAE probes should be mechanically strong, as well as optically and acoustically transparent to maximise the signal strength and minimise any reflection artefacts. A recent study by Iskander-Rizk et al. [112] investigated the acoustic and optical attenuation of several sheath materials at 1718 nm, which corresponds to the location of a prominent lipid optical absorption peak. The results showed that among the other materials in the study, polyethylene (PE) sheath was most suitable for intravascular imaging. Another study by Cao et al. [113] also compared the optical (at 1730 nm) and acoustic attenuation of a series of sheaths made from different materials, showing that polyurethane (PU) was most suitable sheath material owing to its small PA artefact, and large optical and acoustic transmission.

### 3.3 Multimodal PAE probes

Multimodal imaging has been increasingly attractive as it combines the strengths from different imaging modalities with complementary capabilities to provide more comprehensive diagnosis of tissue compared to a single modality. Side-viewing PAE probes usually include pulse-echo US imaging with both modalities sharing the same US transducer. As such, the spatial resolution for the two naturally co-registered modalities in these probes is very similar. In endoscopic applications, the complementary nature of PA and US imaging could be very useful, by superimposing functional and molecular information of tissue provided by PAI and the tissue anatomy visualised by US imaging.

**Fig. 7:**
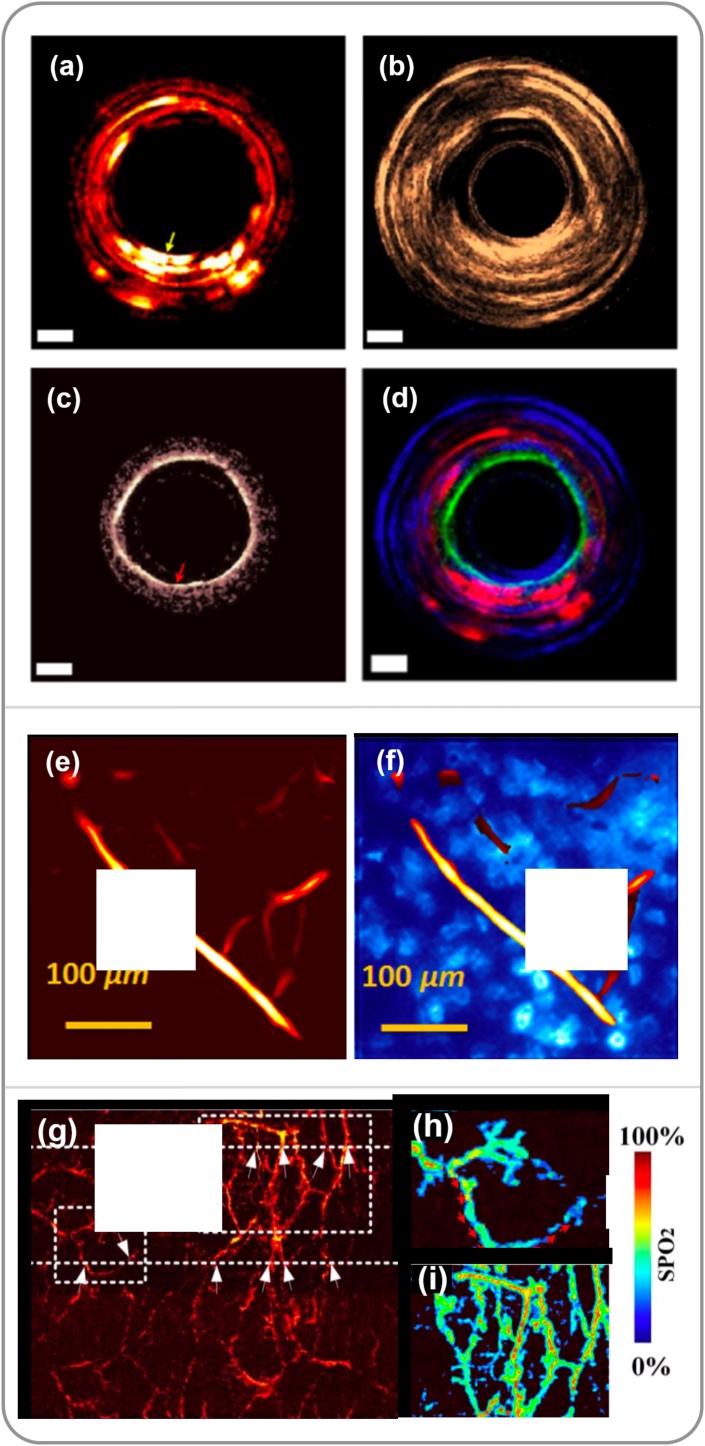
Multimodal endoscopy. (a-d) Tri-modal photoacoustic (PA), ultrasound (US), optical coherence tomography (OCT) imaging of a human artery. (a) PA; (b) US; (c) OCT, and (d) overlay. (a)-(d) was adapted from Ref. [116] with permission. (e) PA image of a mouse ear. (f) Co-registered PA and fluorescence image of a mouse ear at the same location with (e). (e)-(f) was adapted from Ref. [87] with permission. (g) PA image of a rabbit rectum. The blood oxygen saturation in two regions (dash boxes) are shown in corresponding hyperspectral images in (h) and (i). (g)-(i) was adapted from Ref. [118] with permission.

OCT has been integrated into PAE probes to provide microstructural information of tissue [111,[113], [114], [115], [116]]. In 2011, Yang et al. [114] developed a tri-modal PA-OCT-US probe by combining an IVUS probe with two optical fibres; one multimode fibre for delivering PA excitation light and one single-mode fibre for performing OCT imaging, respectively. Concurrent OCT, PA and US imaging was demonstrated with a human ovary *ex vivo*. However, a large probe size of 5 mm limited its interventional use in clinical settings. In 2015, Dai et al. [115] reported a smaller tri-modal probe with 2.3 mm diameter, which employed a single-mode fibre for both PA and OCT, whilst a non-colinear US transducer was used for PA signal detection and performing US imaging. In 2016, the same group [116] reduced the diameter of a tri-modal PA-OCT-US probe to 1 mm, based on a dual-cladding fibre for delivering the PA excitation light through the outer cladding and OCT imaging through the inner single-mode core. Demonstration was performed with imaging of the skin of a human hand *in vivo* and a human artery with an atherosclerotic plaque *ex vivo* (Fig. 7a–d). Most recently, Mathews et al. [111] developed a dual-mode PA-OCT probe based on a single-mode fibre and a fibre-optic FP US sensor.

Other imaging modalities including fluorescence [87,92,117] and hyperspectral microscopy [118] have been integrated into PAE probes to provide additional information. By visualising appropriate fluorescent probes that label specific molecules, fluorescence imaging can complement PAI by providing anatomical and pathological information of tissue and cells. Recently, a number of imaging probes that combined fluorescence imaging with PAE were reported. Typically, PA and fluorescence imaging use the same optical fibre for signal excitation, which also collects the fluorescence light to be detected by a photodetector. In 2012, Shao et al. [87] reported a fluorescence/OR-PAE system based on the use of a fibre bundle for excitation lasers (532 nm for PA and 447.5 nm for fluorescence) delivery. The generated PA signals were detected by an external US transducer, whilst the fluorescence light was collected and transported through the fibre bundle before captured by a camera in front of the proximal end of the fibre bundle. The capability of multimodal imaging was demonstrated by achieving co-registered PA and fluorescence images of a mouse ear (Fig. 7e–f). In 2014, Abran et al. [117] developed a tri-modal PA, US and fluorescence imaging probe. The probe was similar to those non-colinear PAE probes, while two light sources of 710 nm and 780 nm were used for PA and fluorescence signals excitation, respectively. A photomultiplier was placed in front of the proximal fibre end to capture fluorescence light. This design allowed simultaneous imaging at a speed of 30 fps for US and fluorescence imaging, and 1/13 fps for PAI. Hyperspectral imaging is an emerging optical modality that images the rich spectroscopic characteristics of tissue in 2D. In 2017, Liu et al. [118] reported a hyperspectral/PAI system that provided real-time imaging of blood oxygen saturation in addition to PAI (Fig. 7g-i). Briefly, the probe design was based on a colinear side-viewing PAE probe for PAI with excitation light delivered by a single-mode optical fibre and a GRIN-lens. For hyperspectral mode, broadband light was delivered to a liquid crystal tuneable filter that was used as a band-pass filter for wavelength modulation. The light through the filter was coupled into a coherent multicore fibre bundle to illuminate tissue targets. The back-reflected light passed through relay optics and was then detected by a CCD camera placed at the probe proximal end to form hyperspectral images. The capability of both hyperspectral and PA imaging was demonstrated with a mouse ear *in vivo* and a rabbit rectum *in vivo*.

### 3.4 Potential clinical applications of PAE probes

#### 3.4.1 Intravascular photoacoustic imaging

Rupture of an atherosclerotic plaque in the coronary system accounts for the majority of coronary events [[69], [70], [71]], it is therefore very important to accurately identify the vulnerable plaques at an early stage. X-ray angiography has a long history for the evaluation of coronary atherosclerosis, however, it suffers from low soft-tissue contrast and only provides 2D images of coronary arteries for vessel stenosis detection [119,120]. Intravascular US (IVUS) imaging has been used as an adjunct to X-ray angiography, which enables the detection of plaque morphology and location but is usually challenging to differentiate plaque compositions due to its elasticity-based image contrast [121,122]. Intravascular OCT has also been promising to provide microstructural information of vulnerable plaques in coronary arteries, such as thin fibrous cap and endothelium. However, its imaging depth is typically less than 1–2 mm, which limits its ability to characterise the extent of plaque volume [123]. Intravascular photoacoustic (IVPA) imaging is a promising modality for the diagnosis of atherosclerosis by visualising lipid cores of vulnerable plaques. In current IVPA systems, IVUS imaging is usually offered by side-viewing PAE probes, with the US transducer transmitting US waves and receiving both PA signals and pulse-echo US signals, revealing both structural and molecular information of tissues at depths up to a few mm [56,[102], [103], [104], [105],^124^].

**Fig. 8:**
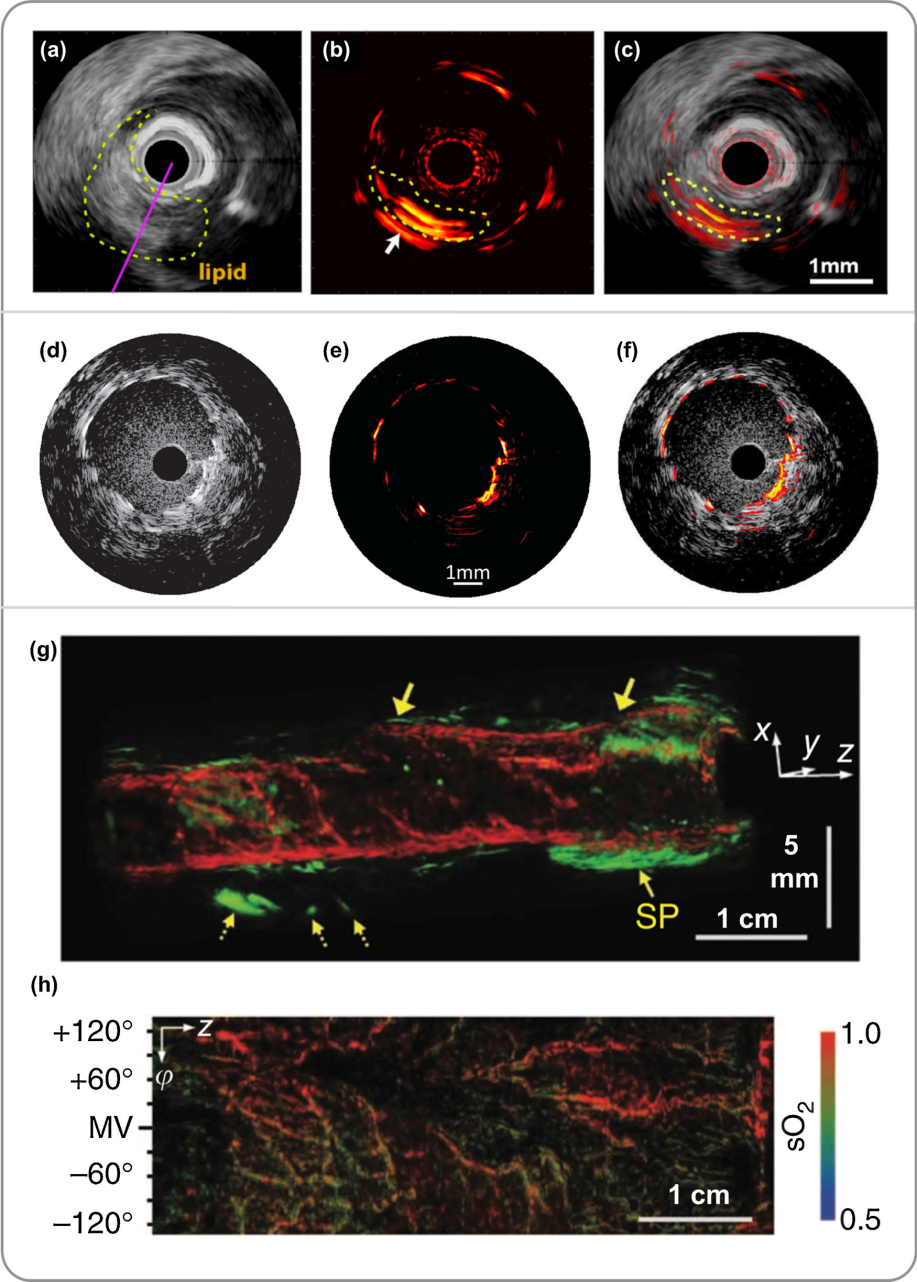
Embodiments of potential clinical applications of photoacoustic endoscopy (PAE) systems. (a-c) Cross-sectional ultrasound (US), photoacoustic (PA), and PA and US overlay images of a swine coronary artery *in vivo*. PAI clearly visualised a lipid core (in yellow circle). (a)-(c) was adapted from Ref. [126] with permission. (d-f) Cross-sectional US, PA, and PA and US overlay images of a vascular stent of rabbit thoracic artery *in vivo*. (d)-(f) was adapted from Ref. [127] with permission. (g) co-registered 3D PA (red) and US (green) image of a rat colon. The dotted arrows marked mesenteric tissue entangled around the tract. SP, sphincter. Horizontal scale bar, 1 cm; vertical scale bar, 5 mm. (h) functional image of the blood oxygen saturation level from the inside of the colon shown in (g). (g)-(h) was adapted from Ref. [57] with permission.

In the past decade, a few research groups have obtained promising results from studies on *ex vivo* human arteries with atherosclerosis plaques [51,52,64,98,99]. In 2011, Jansen et al. [56] reported the first study on spectroscopic IVPA imaging of human coronary *ex vivo* with a side-viewing PAE probe. Lipid within the plaques was differentiated from the surrounding tissue with excitation at 1210 nm. In 2012, Wang et al. [125] developed a PAE probe for IVPA imaging at 1720 nm provided by an optical parametric oscillator (OPO) system, where lipid exhibits another absorption peak. *In vivo* imaging was demonstrated on a rabbit aorta, which suggested that IVPA imaging at this wavelength without blood flushing is feasible. In 2014, Jasen et al. [23] compared the 1.2 μm and 1.7 μm wavelength bands, reporting that both of them are suitable for lipid identification in IVPA imaging. Real-time IVPA imaging, which is required for clinical use, was realised by using laser sources with high PRFs. In 2017, Wu et al. [126] developed a multispectral IVPA imaging system with a wavelength range of 1700–1750 nm by using a periodically-poled LiNbO_3_ OPO pumped by a pulsed Nd:YAG laser as the excitation source. With a PRF of 5 kHz, it allowed image acquisition at 20 fps, which was demonstrated with a swine coronary artery *in vivo* and human coronary artery *ex vivo* (Fig. 8a–c). In the same year, Hui et al. [104] reported a real-time IVPA imaging system for rabbit aortas *in vivo* and human coronary arteries *ex vivo*. The laser source in this work was a 1700 nm master oscillator power amplifier-pumped OPO with a PRF of 2 kHz. Both these works demonstrated the capability of real-time IVPA imaging for atherosclerosis diagnosis. Besides plaque identification, IVPA was also demonstrated for guiding minimally invasive procedures such as stent deployment with phantoms and *ex vivo* tissue samples [55,61,127,128]. In 2012, Karpiouk et al. [127] developed a side-viewing PAE imaging system and demonstrated the visualisation of stent in a rabbit thoracic artery. With co-registered US and PA images, the structure of vessel walls was visualised in US, whilst the metal stents were highlighted with PAI owing to its strong optical absorption (Fig. 8d–f). In 2017, VanderLaan et al. [128] reported a real-time IVPA/US probe operating at 33 fps and validated it on a vessel-mimicking phantom with an embedded coronary stent.

#### 3.4.2 Gastrointestinal tract photoacoustic imaging

Imaging in the gastrointestinal tract has attracted intensive research interest, mainly for the detection of early-stage tumour located in or close to the tract walls, such as oesophageal cancer [129,130] and colorectal cancer [57,98,131,132]. PAE holds great promise to visualise changes in vascular morphology and blood oxygenation and metabolism that are known to be associated with tumour development. In the past decade, a series of side-viewing PAE imaging systems have been developed and validated on small animal models, including rat rectum *in vivo* [131], melanoma tumour in rat colorectum *in vivo* [132], tumours of oesophagus and reflux esophagitis of a rabbit [129]. Visualisation of vascular morphology in the gastrointestinal tract was demonstrated with co-registered PA and US images. In 2012, Yang et al. [57] developed a PAE system and demonstrated its capability of visualising both the gastrointestinal tract walls and surrounding internal organs *in vivo*. Furthermore, the employment of multiwavelength laser sources enabled the measurement of blood oxygen saturation (Fig. 8g-h), which functionally differentiated aorta and caudal vena cava near a rabbit oesophagus, and stained the lymphatic system surrounding a rat colon.

## 4 Future perspectives

In this section, we present our thoughts on future directions of technological development that could pave the way for clinical translation of miPAI.

### 4.1 Light sources

Despite that tremendous potential has been demonstrated, widespread clinical adoptions of current miPAI systems has been slowed down by the available light sources for PA excitation. Currently, Q-switched solid-state lasers with a single wavelength were commonly used for vascular (532 nm) and stent imaging (at 1064 nm). Q-switch pumped OPO systems were used to provide optical excitation at a wide range of wavelengths including 1210 nm and 1720 nm for lipid imaging. However, apart from the large dimensions and high costs, the low PRF and speed of wavelength-tuning of the OPO systems significantly restricted the imaging speed of current systems. Thus, novel light sources with fast wavelength-tuning, high output pulse energy and fast PRF are highly desired for clinical translation of miPAI.

Recently, laser diodes (LD) and light emitting diodes (LED) have been studied as promising alternatives to solid-state laser sources in PAI [[133], [134], [135]]. Compared with solid-state lasers, these light sources exhibited benefits in low cost, small sizes, and high PRF (tens of kHz) with a wide range of wavelengths. These advantages make LDs and LEDs well suited for real-time PAI, especially in a clinical environment. However, LD or LED light sources usually suffer from low output energies, which could lead to low signal-to-noise ratios (SNRs). This limitation can be mitigated in the following ways. First, as the PRF of these light sources are high, averaging across multiple signal acquisitions can improve the SNR. Second, the SNR can be further improved by pulsed light with coded excitation [136] or intensity modulated continuous-wave (CW) light with a chirped frequency [137]. With the use of these light sources, various PAM and PACT systems have been demonstrated with imaging of red blood cells [138], vasculatures [[139], [140], [141], [142]], melanoma tumour [143], invasive devices [144,145] and atherosclerotic plaques [146,147]. Further, LDs and LEDs have been promising for use in miPAI systems as they can be easily miniaturised to mm level and fabricated in multi-element curved arrays, which could facilitate the probe design. With low-cost, and compactness, LDs and LEDs could be a next generation PA excitation light sources in clinical real-time miPAI systems.

### 4.2 All-optical imaging

In recent years, optical micro-resonator-based US sensors have attracted significant research interests [[148], [149], [150], [151]]. This is because of a number of distinct advantages that are associated with these US sensors compared to conventional piezoelectric US transducer. First, optical US sensors are immune to electromagnetic interferences, which makes them compatible with intra-operative MRI for guiding surgical procedures. Second, due to their transparent nature, optical US sensor can be placed in the light path for excitation laser delivery, which could allow for co-linear designs of the light delivery and US detection, to facilitate the integration and miniaturisation of the probe and maximise the SNR. Third, some of the optical US detectors are sensitive, broadband and can be readily miniaturised without sacrificing their acoustic sensitivity and bandwidth. In contrast, the acoustic sensitivity of piezoelectric US detectors decreases with the active surface areas, which becomes problematic with miniature detectors as these degrade the SNRs of images. While polyvinylidene difluoride (PVDF) detectors can have wide bandwidth, their sensitivity is usually lower compared to piezoceramic/piezocomposites detectors due to smaller electromechanical coupling coefficients [152]. However, the latter have narrower detection bandwidths, especially with high frequency miniature detectors, which worsen the spatial resolution of images [153]. Therefore, optical US sensors hold great potential for developing miniature PA probes. In the past decade, Beard et al. have pioneered the development of sensitive and broadband fibre-optic FP US detectors [[94], [95], [96]]. Other types of optical US sensors have also been investigated for PAE probes, including micro-ring resonators [108,109] and π-phase shifted fibre Bragg gratings [154].

All-optical US imaging could be useful in PAE probes to provide complementary structural information of tissue in many applications, in which, US was generated with optically absorbing materials coated on a fibre tip by pulsed light illumination via the PA effect [[155], [156], [157]]. Pulse-echo signals from tissue can be recorded with an optical ultrasound detector for US imaging. Most recently, Noimark et al. [157] developed an all-optical dual-mode PA and US probe, which comprised a multimode optical fibre with gold nanoparticles- or dye-based composites coated at its distal end, and a fibre-optic FP US sensor. Owing to the narrow but strong optical absorption bands, the coatings generated broadband US waves at the fibre tip for US imaging with optical excitation at 532 nm, while allowed the transmission of light at 1210 nm for PAI of lipid contrast. Images of diseased human aorta tissue *ex vivo* were acquired by linear translation of the probe.

### 4.3 PA sensing

In additional to intraoperative imaging, point-based PA spectroscopic sensing could be useful for guiding minimally invasive procedures by identifying procedure targets and avoiding damaging of critical tissue structures such as major blood vessels during tumour resections and peripheral nerves during nerve blocks. In 2016, Mathews et al. [158] developed a miniature fibre-optic probe for minimally invasive PA sensing. A single optical fibre was used to deliver excitation laser for the generation of PA signals, whilst a dichroic FP US sensor was integrated at the fibre tip to receive these US waves. The interrogation light for the US sensor was also transported through the same fibre. PA signals from a blood vessel mimicking phantom were detected at a distance up to 7 mm in front of the fibre tip. In 2015, Xia et al. [159] reported a dual-mode fibre-optic PA and US sensing probe for guiding minimally invasive procedures. The probe comprised three optical fibres; one multimode fibre for delivering pulsed excitation light to tissue, a second multimode fibre with an optical absorbing coating for transmitting US, and a third single-mode fibre with a FP cavity at the distal end for receiving US. In this work, in addition to the PA/US sensing of a phantom in front of the probe tip, the fibre-optic US sensor was also used to communicate with an external clinical US array to identify the tip of the probe. This US-based medical device tracking method could be useful in many minimally invasive procedures by accurate identification of the interventional device at sub-millimetre level [160,161,162,163,164]. Although PA sensing is at early stages of development, it allows depth-resolved spectroscopic tissue contrast to be acquired at depths well beyond those for optical spectroscopy methods, and thus it holds the potential to provide intra-operative guidance during surgical and interventional procedures.

In summary, PAI has undergone exponential growth in the past decade and has emerged as one of the most exciting areas in biomedical imaging. With promising preclinical and clinical demonstrations, translating PAI into clinical practice has attracted significant interest. In particular, miPAI that enables PA examinations of deep tissues with internal light illuminations has significantly extended the clinical applicability of PAI. With continuous technical breakthroughs such as those in novel light sources and US detectors, we expect that miPAI could be useful in many clinical disciplines in the future.

## Declaration of Competing Interest

The authors declare that there are no conflicts of interest. A.E.D. is a Director and Shareholder of Echopoint Medical, London, UK, and T.V. holds shares from Mauna Kea Technologies, Paris, France, which, however, did not support this work.
